# Effectiveness of Efflux Pump Inhibitors as Biofilm Disruptors and Resistance Breakers in Gram-Negative (ESKAPEE) Bacteria

**DOI:** 10.3390/antibiotics8040229

**Published:** 2019-11-19

**Authors:** Akif Reza, J. Mark Sutton, Khondaker Miraz Rahman

**Affiliations:** 1 Institute of Pharmaceutical Science, King’s College London, London, SE1 9NH, UK; akif.reza@kcl.ac.uk; 2 National Infections Service, Public Health England, Porton Down, Salisbury, Wiltshire SP4 0JG, UK; mark.sutton@phe.gov.uk

**Keywords:** antimicrobial resistance, biofilm, efflux pump inhibitors, antibiotic potentiation, ESKAPEE pathogens, Gram-negative bacteria

## Abstract

Antibiotic resistance represents a significant threat to the modern healthcare provision. The ESKAPEE pathogens (*Enterococcus faecium.*, *Staphylococcus aureus*, *Klebsiella pneumoniae*, *Acinetobacter baumannii*, *Pseudomonas aeruginosa*, *Enterobacter* spp. and *Escherichia coli*), in particular, have proven to be especially challenging to treat, due to their intrinsic and acquired ability to rapidly develop resistance mechanisms in response to environmental threats. The development of biofilm has been characterised as an essential contributing factor towards antimicrobial-resistance and tolerance. Several studies have implicated the involvement of efflux pumps in antibiotic resistance, both directly, via drug extrusion and indirectly, through the formation of biofilm. As a result, the underlying mechanism of these pumps has attracted considerable interest due to the potential of targeting these protein structures and developing novel adjunct therapies. Subsequent investigations have revealed the ability of efflux pump-inhibitors (EPIs) to block drug-extrusion and disrupt biofilm formation, thereby, potentiating antibiotics and reversing resistance of pathogen towards them. This review will discuss the potential of EPIs as a possible solution to antimicrobial resistance, examining different challenges to the design of these compounds, with an emphasis on Gram-negative ESKAPEE pathogens.

## 1. Background

Antimicrobial resistance (AMR) is a natural phenomenon and an intrinsic property of bacteria that occurs upon exposure to antibiotics due to their genetic flexibility and adaptability. However, over the years, this process has been grossly accelerated through the overuse, inappropriate prescribing and extensive agricultural use of antibiotics and other antimicrobial agents. This process, termed acquired resistance, has resulted in the emergence of multi-drug resistant (MDR) bacteria. The global burden of bacterial infections due to AMR has grown at an alarming pace. According to an exhaustive review commissioned by the UK government, globally, approximately 700,000 deaths can be attributed to AMR each year [1]. Furthermore, based on increasing incidence of drug resistance among bacterial infections, this toll is expected to exceed 10 million by 2050, at a cumulative global cost of 100 trillion US dollars (USD). Additionally, global surveillance data from the WHO reported a widespread occurrence of AMR among 500,000 people with suspected bacterial infections across 22 countries (ranging from high to low-income) [2]. Notably, resistance to penicillin, a *β*-lactam antibiotic widely used for decades to treat a range of different bacterial infections, was reported amongst 51% of the surveyed countries. However, despite the apparent exacerbation of this global issue, the lack of any new classes of antibiotics coupled with the inevitable emergence of bacterial resistance have caused antibiotic development to lack economic appeal to the pharmaceutical industry. Evidently, of 38 billion USD invested into pharmaceutical research and development between 2003–2013, only 1.8 billion USD was invested into AMR research [1].

**Figure 1:**
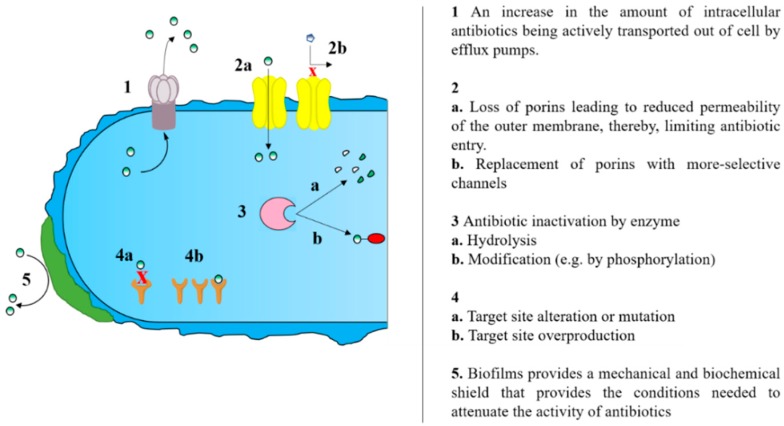
Schematic diagram of the different mechanisms facilitating acquired resistance.

Acquired resistance can be caused by horizontal gene transfer (HGT), or spontaneous chromosomal mutations, or a combination of both factors [3,4]. HGT, one of the predominant driving force of multi-drug resistance rise, is the transfer of resistance gene between bacteria through the use of exogenous genes: Plasmids, transposons and integrons [4]. The underlying biochemical basis for AMR include several mechanisms such as drug inactivation/alteration through hydrolysis or other modifications; alteration or overexpression of drug binding site; porin loss resulting in limited passage of antibiotic in cell; increased efflux of antibiotics through pumps; and biofilm formation [4,5] (Figure 1).

Biofilms are defined as ‘aggregates of sessile microorganisms in which cells are embedded in an autogenous matrix of extracellular polymeric substances (EPS) that are irreversibly attached to a substratum or interface or each other’ [6]. They are typically found on wet, surfaces found in natural, clinical and industrial settings [7]. Their prevalence on clinical settings including medical devices and/or on dead/living tissue (e.g., sequestra of dead bone, heart valves, prostheses, contact lenses, intestines, lungs and tooth enamel [8] has been shown to be a major contributor to a number of different nosocomial infections [9]. Furthermore, the antibodies released in response to biofilms have also been shown to induce immune complex damage to neighbouring tissues [10].

Biofilm formation is initiated by several environmental factors including mechanical signals, nutritional and metabolic cues, host-derived signals, sub-inhibitory concentration of antimicrobials and quorum signals (QS) [11]. One of the key roles of this process is to act as a major defence mechanism against internal and external threats. This, in turn facilitates their greater resistance and tolerance to antibiotics and disinfectants, in comparison to planktonic organisms. Biofilms achieve this through several mechanisms including diminished permeability of antibiotics through the EPS matrix [12], physiological changes induced by reduced growth rate and starvation response (nutrient and oxygen deprivation) [13], increased expression of efflux pumps [14] and the emergence of persister cells (a wild-type cell able to resist antimicrobial effects) within the biofilm [15].

Biofilm-associated infections are extremely recalcitrant to antimicrobial therapy due to their propensity to resistance and have consequently rendered antibiotic monotherapies inadequate. Moreover, their robustness has enabled certain species of bacteria to develop both genetic and phenotypic resistance to a range of antibacterial therapies. For instance, despite intensive treatment, *Pseudomonas aeruginosa* (a biofilm producer) related infections have been shown to persist in cystic fibrosis (CF) patients [16]. Their persistence enables them to successfully disperse planktonic cells and promote biofilm formation in the body which can cause chronic infections (Table 1). In addition to the ESKAPEE pathogens (*Enterococcus faecium., Staphylococcus aureus, Klebsiella pneumoniae, Acinetobacter baumannii, Pseudomonas aeruginosa, Enterobacter spp.* and *Escherichia coli*), biofilm production has been reported to be prevalent in Gram-positive (*Bacillus* spp., *Listeria monocytogenes* and lactic acid bacteria [17]) and other Gram-negative bacteria (*Klebsiella oxytoca*, *Proteus vulgaris*, *Proteus mirabilis*, and *Morganella morganii* [18]).

## 2. Efflux Pump Inhibitors and Antibiotic Potentiation

**Figure 2:**
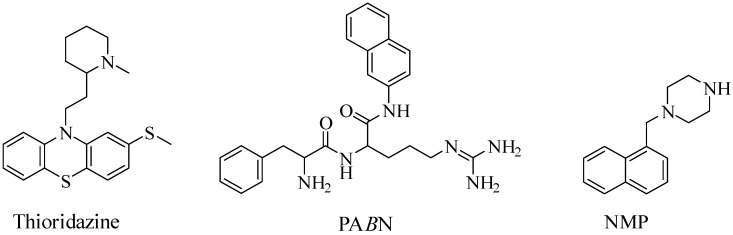
Chemical structures of the inhibitors used by Kvist et al [35].

Studies have demonstrated the influential role of efflux pumps in the formation of bacterial biofilm. In a majority of these studies, the expression of such pumps is shown to be upregulated in biofilms, which confers increased resistance against antibiotics. Kvist et al [35] conducted a pivotal study, demonstrating the importance of efflux pumps in bacterial biofilms; thus establishing the potential of EPIs as anti-biofilm agents. The effects of three known EPIs, PA*β*N, thioridazine and NMP (Figure 2) were tested against *S. aureus*, *K. pneumoniae, P. aeruginosa* and *E. coli*. The inhibitors caused a significant reduction in biofilm formation in almost all of the strains—unlike the other two inhibitors, NMP did not result in any noticeable reduction in *S. aureus* biofilm. In addition, the EPIs were shown to enhance the antibacterial activity of antimicrobial agents. To confirm the role of the efflux pumps in biofilm production, tests were conducted using mutants and wild type strains of each bacterial species. The direct effect of EPI on biofilm mass was shown to be a good indicator of the role of the antimicrobial agent on biofilm disruption [36]. Several other studies have demonstrated the role of efflux pumps in the formation of biofilms in individual ESKAPEE pathogens.

**Figure 3:**
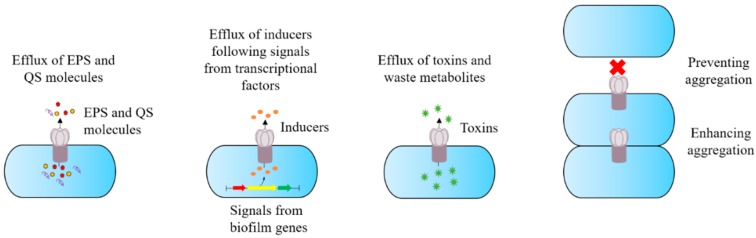
Schematic diagram of the potential role of efflux pumps in biofilm formation, as demonstrated through numerous studies. Adapted from Alav et al. [35]. EPS: extracellular polymeric substance; QS: quorum signals.

Inhibiting or down regulating these efflux pumps have provided a promising new approach to decelerate and/or prevent the emergence of antibiotic resistance. The exact role of efflux pumps in the development and maintenance of biofilms differs between bacterial species. Nevertheless, the mechanisms behind their contribution have been subdivided into four categories: efflux of molecules required for biofilm formation (EPSs) and regulation (molecules associated to the quorum system); indirect regulation of transcriptional factors involved in biofilm formation; efflux of toxins (e.g., antibiotics) and waste metabolites; and facilitating aggregation by influencing adhesion to both cells and other surfaces [37] (Figure 3). Other potential mechanisms may also exist.

For EPIs to be clinically relevant, it must show therapeutic activity at an attainable serum/tissue sample with minimum toxicity. As combination therapies, EPIs must function synergistically with their co-administered antibiotics and achieve a greater effectiveness than that achieved by the individual agents alone. If the effect of the EPI in modifying biofilm formation is the primary effect solely attributed to the inhibition of the target pumps [38], then the EPIs must show adequate potency to potentiate this activity.

Despite, the promise shown from the numerous studies centred on EPIs, the emergence of an approved, potent EPI has proven to be a herculean task. This can be attributed to a range of factors, including the structural heterogeneity of EPIs, broad multi-drug resistance efflux pump substrate specificity and off-target toxicity [38]. A majority of the investigational EPIs are often discovered serendipitously while screening large libraries of compounds with in vitro pump inhibitory activity. Evidently, high-throughput screening (HTS) and structure–activity relationship (SAR) studies remain the best approach to identify new EPIs. Moreover, due to the significance of efflux pumps in ESKAPEE pathogens, the application of EPIs has been the focus of current research efforts to combat AMR among these organisms.

## 3. *Klebsiella pneumoniae*

*K. pneumoniae* is a non-fastidious, Gram-negative bacillus, encapsulated, nonmotile, rod-shaped member of the *Enterobacteriaceae* family, frequently found in a variety of environmental niches including water bodies such as the drinking-water distribution system, soil and vegetation [39]. *K. pneumoniae* has emerged as a clinically significant healthcare-associated pathogen; it is a causative agent in approximately 14–20% of respiratory tract, lower biliary duct, surgical wounds and urinary tract-related infections (UTI) [40]. Recently, the bacteria have acquired a large variety of *β*-lactamase enzymes, thus, enabling the degradation of *β*-lactam antibiotics such as penicillin, cephalosporins, and carbapenems [40]. Because carbapenems are conventionally used as the last resort for the treatment of infections caused by Gram-negative, carbapenem-resistant *K. *p*neumoniae* (CRKP) is a significant problem in the medical domain [41]. Similarly, to other pathogens, *K. pneumoniae* biofilms enhances their persistence on epithelial tissues and medical device surfaces, and acts as a protective barrier against antimicrobial agents [40]. Their ability to form biofilm has been a major contributing factor to UTI, which is one of the most prevalent type of nosocomial infection caused by the pathogen [40].

### EPIs and Their Potential Role in Biofilm Disruption in K. Pneumoniae

**Figure 4:**
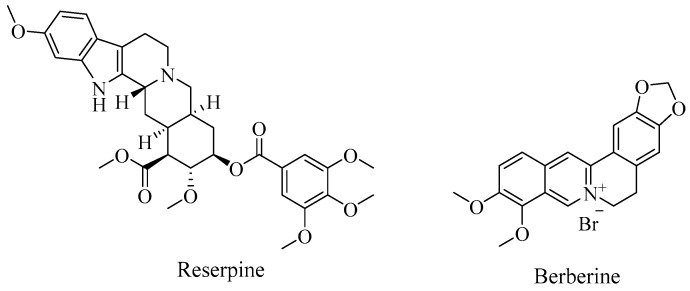
Chemical structures of the plant-derived alkaloids, reserpine and berberine.

The involvement of the AcrAB pump in *K. pneumoniae* biofilm formation [39], suggests that inhibition of the efflux pump system might potentiate the disruption of biofilm formation. It has been long reported that the natural alkaloids, reserpine and berberine (Figure 4) can potentially inhibit a diverse range of different efflux pumps, including the Resistance Nodulation Division (RND) family [42]. In a comprehensive study, Magesh et al [42]. evaluated the effects of six different natural compounds on the MIC and biofilm inhibition in strong biofilm forming *K. pneumoniae* isolates. Reserpine was identified as the more potent and effective EPI—the alkaloid exhibited a relatively low minimum biofilm inhibition concentration (MBIC) with respect to the other substances. Reserpine has shown neurotoxic properties at high concentration; consequently, the low MBIC provided further promise of using the EPI in combination with antibiotics [42]. Berberine, while not as effective alone, has been shown to potentiate ciprofloxacin against MDR *K. pneumoniae* isolates [43]. The MIC of ciprofloxacin (shown to be ineffective against *K. pneumoniae* biofilm [12]) was reduced by berberine compared with either of the single agents. The reduced MIC enabled the antibiotic to arrest bacterial growth and prevent biofilm formation in vitro. Due to the observations of this study, berberine merits further in vivo study as an antibiotic potentiator.

One of the most studied anti-efflux compound in *K. pneumoniae* is PA*β*N (Figure 2), a competitive EPI, that is considered a substrate to a broad-spectrum of pumps [35]. The EPI has been reported to significantly reduce the MIC of quinolones, chloramphenicol and tetracycline (AcrAB substrates) in MDR *K. pneumoniae* strains [44]. This has been speculated to be a result of the competitive nature of PAβN—the compound outcompetes the antibiotic substrates; thereby, increasing their intracellular concentration. One of the main concerns regarding this compound is its toxicity and low stability, which has limited its clinical potential. As will be discussed later, derivatives of this compound have been tested on other Gram-negative bacteria including *P. aeruginosa* and *E. coli* [45].

**Figure 5:**
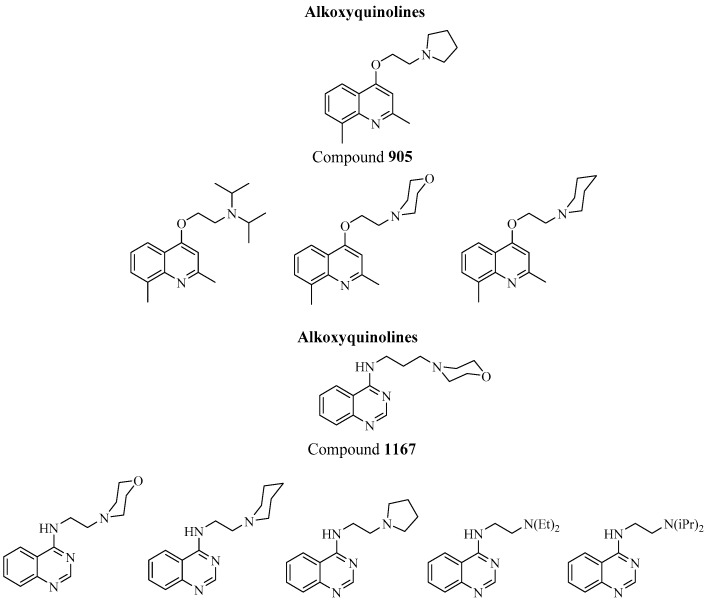
Chemical structures of some of the efflux pump-inhibitors (EPIs) investigated for potential use against *K. pneumoniae*.

Several different quinoline derivates have been studied for their ability to restore the activity of efflux-effected antibiotics [44]. One such class of synthetic anti-efflux compounds, with reported activity against AcrAB pumps, are alkoxyquinolines. Chevalier et al. [46] studied a group of different alkoxyquinoline derivatives to investigate their ability to restore antibiotic susceptibility to resistant clinical isolates of *Enterobacter aerogenes* and *K. pneumoniae*. Mutant *K. pneumoniae* isolates (KP55) exhibiting active efflux of norfloxacin and chloramphenicol, as well as mutant isolates of *E. aerogenes* (EA27) overexpressing AcrAB pumps were used for the study. Compound 905, in particular, (Figure 5), was able to significantly reduce its MIC in both isolates via AcrAB-TolC inhibition. Similarly, Mahamoud et al. [45] reported alkylaminoquinazoline derivates (Figure 5), able to restore antibiotic activity in Gram-negative resistant isolates, including *K. pneumoniae*. Among these, compound 1167 (Figure 5) increased the activity of chloramphenicol, nalidixic acid and tigecycline (RND substrates) susceptibilities towards resistant strains, with respect to the control (PA*β*N). In a different study, quinazoline derivatives were evaluated as chemosensitizers of antibiotic activity in resistant strains of *E. aerogenes*, *K. pneumoniae* and *P. aeruginosa* [47]. The compounds were able to restore the intracellular concentration and increase the susceptibility of chloramphenicol and nalidixic acid in isolates of the three Gram-negative bacteria, overexpressing efflux mechanisms [47].

## 4. *Acinetobacter Baumannii*

*A. baumannii* is an opportunistic, aerobic, non-motile Gram-negative bacillus that has recently exhibited high incidence among immunocompromised individuals and is increasingly becoming prevalent in patients experiencing prolonged hospital-stay [48]. While the bacteria are usually found in hospital environments, they have also been reported to colonise the skin, as well the respiratory and oropharynx secretions of infected individuals [48]. Over the last 15 years, MDR *A. baumannii* has emerged as a leading cause of infections contracted in long-term care facilities and in wounded military personnel, due to its remarkable ability to acquire resistant determinants. Recently, according to the WHO, carbapenem-resistant *A. baumannii* was reported as one of the MDR pathogens posing the greatest threat to the human health, further highlighting the need for novel antibiotics [41]. It has been reported to contribute to various nosocomial infections including pneumonia, bacteraemia, endocarditis, skin/soft tissue infections, urinary tract infections, and meningitis, among others. Furthermore, the pathogen frequently causes biofilm-related infections including ventilator-associated infections and catheter-related infections [37].

### EPIs and Their Potential Role in Biofilm Disruption in A. Baumannii

**Figure 6:**
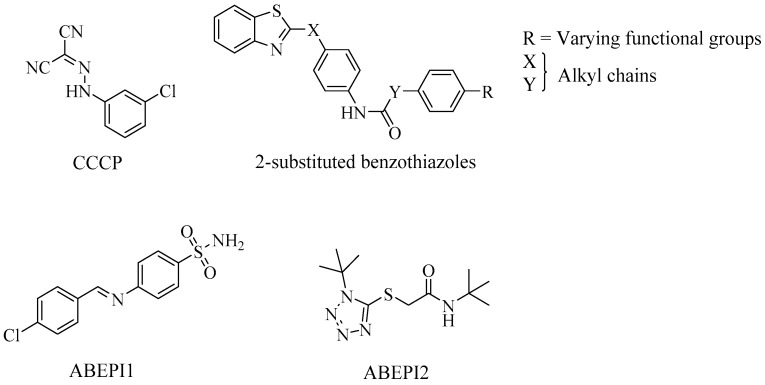
Investigational EPIs tested against *A. baumannii*.

Despite the significance of the efflux system in *A. baumannii*, studies on EPIs are currently limited against this pathogen. Nevertheless, a few putative bacterial EPIs have been described including PA*β*N, 1-(1-naphthylmethyl)-piperazine (NMP) (Figure 2) and carbonyl cyanide 3-chlorophenylhydrazone (CCCP) (Figure 6), three of the most studied synthetic inhibitors in *A. baumannii*. Using the known EPIs, PA*β*N and NMP, Pannek et al. [49] investigated the ability of the inhibitors to reverse multi-drug resistance in *A. baumannii* strains. PA*β*N, demonstrated inhibitory activity against the AdeFGH pumps by reducing the MIC of trimethoprim, chloramphenicol and clindamycin (AdeFGH substrates) [50]. However, its inability to reduce rifampicin MIC (also an AdeABC and AdeFGH substrate), indicated the presence of two different efflux mechanisms, one of which was independent of PA*β*N-mediated inhibition [49]. At a higher concentration, the aryl-piperazine derivative, NMP, was more active, enabling, a significant reduction in the MIC of fluoroquinolones and aminoglycosides (AdeABC substrates). Furthermore, this observation was not limited to strains overexpressing AdeABC, thereby suggesting the presence of another mechanism. Both EPIs partially reversed multi-drug resistance in *A. baumannii*, however, there are strong indications of the presence of a different mechanism of action, independent of the RND efflux system [49]. CCCP is a synthetic inhibitor of proton motive-force-dependant pumps, including RND pumps, that has often increased the susceptibility of various MDR bacteria, including *A. baumannii*, to different antibiotics [50,51]. In a cohort study, 65 *A. baumannii* strains were isolated from hospitalised burn patients—in 86% of the patients, exposure to CCCP reduced the MIC of ciprofloxacin (RND substrate) by 2–64 fold [52]. This was suggestive of the role of the efflux-based system in ciprofloxacin resistance in the pathogen and further highlights the ability of CCCP to inhibit the previously mentioned efflux pumps. The EPI has also increased the susceptibility to other substrates including imipenem and colistin [53,54].

Recently, Runci et al. [55] exhibited the ability of the human serum to support both the planktonic and biofilm growth of *A*. *baumannii*. This represents a major concern for clinicians, due to the invasive nature of the subsequent treatment. Blanchard et al. [56] identified two novel serum-associated EPIs that potentiated the activities of antibiotics toward serum-grown *A. baumannii*, using HTS: ABEPI1 and ABEPI2. Both compounds exhibited similar antibiotic potentiation-profiles towards minocycline and ciprofloxacin and limited the efflux properties of the pathogen in standard Ethidium Bromide (EtBr) assays. EtBr is commonly used to quantify the extent of efflux inhibition in efflux assays as it is an efficient substrate of a wide range of efflux pumps [57]. Furthermore, neither compounds showed cytotoxicity towards human cells, which has limited EPI development in the past. Additionally, the compounds also demonstrated inhibitory activity in *P. aeruginosa* strains. Following SAR, it was speculated that the compounds offer scaffolds amenable to potential alterations to provide analogues aimed at combating MDR Gram-negative bacteria [56]. Yilmaz et al. [58] generated a range of different pharmacophore models consisting of 2-substituted benzothiazoles, in silico. SAR analysis of the compounds revealed that the conformational properties of the compounds are significant for inhibition of AdeABC efflux pumps. This was further tested in vitro using the AdeABC-overexpressing clinical isolate of *A. baumannii*, SbMox-2. Although no significant antibacterial activity was observed when tested alone, in combination, the pharmacophores significantly reduced the MIC of ciprofloxacin, and subsequently, reversed its antibiotic activity against SbMox-2.

## 5. *Pseudomonas Aeruginosa*

*P. aeruginosa* is a ubiquitous Gram-negative bacterium, able to colonise a wide range of different environmental niches including soil and large bodies of water, due to its adaptability [37]. Clinically, it is a leading cause of nosocomial infections among immunocompromised patients and chronic infections in cystic fibrosis (CF) patients. This can be attributed to its intrinsic and acquired ability to exhibit resistance to different classes of antibiotics. Notably, carbapenem-resistant *P. aeruginosa* has been designated as the second most threatening MDR pathogen to the human health, by the WHO [41]. The intrinsic resistance is more prominent in biofilm producing *P.* aeruginosa; which further limits the availability of therapeutic options against *P. aeruginosa* [37].

### 5.1. EPIs and Their Potential Role in Biofilm Disruption in P. Aeruginosa

**Figure 7:**
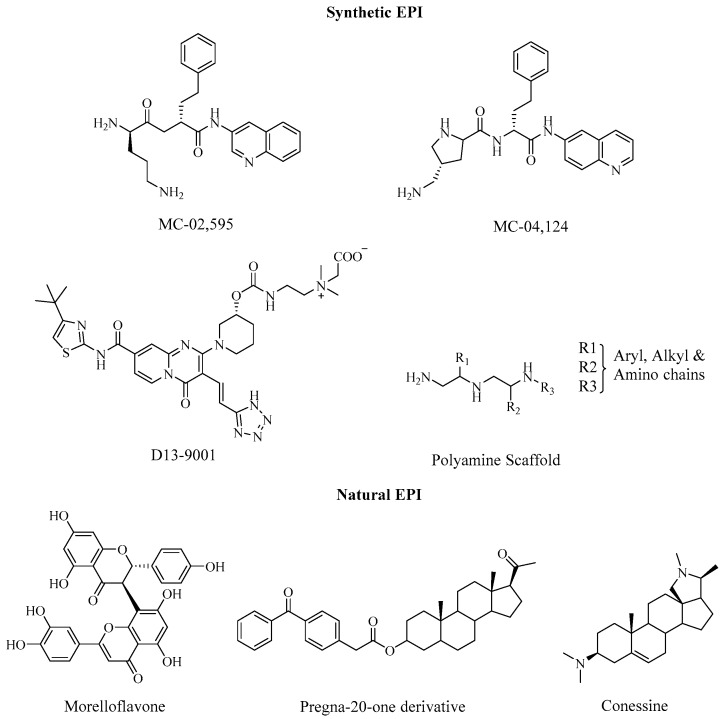
EPIs directed against Mex efflux pumps in *P. aeruginosa*.

Over the last few years, various compounds have been synthesised and investigated for their inhibitory activity against *P. aeruginosa* efflux pumps. Peptidomimetics, including the pioneer lead compound PAβN (MC-207,110), is currently one of the most extensively studied compounds as EPIs against the *P. aeruginosa* efflux system. PA*β*N (Figure 2), a broad-spectrum EPI, have been shown to potentiate of the fluoroquinolones, levofloxacin, by outcompeting the antibiotics for the pumps [59]. Lomovskaya et al. [60] reported the screening and identification of a series of peptidomimetics, with the ability to block fluoroquinolone efflux in MDR *P. aeruginosa* strains (overexpressing MexAB). Notably, MC-02,595 (Figure 7), one of the earliest derivatives of PA*β*N, reduced the MIC of levofloxacin eight-fold, in PAM-1032, a *P. aeruginosa* mutant, over-expressing the MexAB-OprM pump [61]. The EPI maintained all the favourable biological features while attaining higher stability in both murine and human serums. The optimisation of the two peptidomimetics resulted in MC-04,124, which exhibited similar potency and reduced toxicity [62]. MC-014,124 also reduced the MIC of levofloxacin in PAM-1032 strains. Furthermore, analogues of the peptidomimetic retained broad-spectrum pump inhibitory characteristics of the entire *P. aeruginosa* Mex series, as evident, from the blockade of MexAB-OprM, MexCD-OprJ and MexEF-OprN RND efflux pumps.

Yoshida et al. [63] generated and characterised a series of pyridopyrimidine analogues, by incorporating hydrophilic substituents onto the aryl core. Among them, D13-9001 (Figure 7), a morpholine analogue, retained good in vitro activity, while potentiating levofloxacin and aztreonam activity in vivo, in animal models infected with *P. aeruginosa* mutants (overexpressing MexAB-OprN). The compound and its derivatives exhibited an excellent solubility and a favourable safety profile. Moreover, D13-9001 has been shown to significantly reduce the invasiveness of *P. aeruginosa* mutants (overexpressing MexAB-OprN), in comparison with PA*β*N, further highlighting the potential of the pyridopyrimidine to manage *P. aeruginosa* virulence [64]. However, unlike PA*β*N, D13-9001 had no influence on *P. aeruginosa* growth, possibly due to its reduced cytotoxicity.

Liu et al. [65] paired PA*β*N with the iron chelators acetohydroxamic acid, EDTA and 2,2-dipyridyl to assess their synergistic activities against *P. aeruginosa* and biofilm growth. The combination of EDTA and PA*β*N resulted in the largest decrease in biofilm mass (2.5-fold) compared with the PA*β*N alone. Iron functions as an essential signal during *P. aeruginosa* biofilm formation, and consequently, iron chelators disrupt biofilm formation by reducing the available iron concentration in the pathogen [37]. The pyranopyridine series, MBX2319, a known inhibitor of the AcrAB pump (*E. coli*), has exhibited potent efflux-inhibitory activity in the presence of Polymyxin B nonapeptide (PMBN) [66]. The EPI will be further discussed under *E. coli*, due to its significance in the bacteria.

Using HTS, Fleeman et al. [67] identified five different polyamine derivatives, consisting of a core scaffold (Figure 7) with EPI activity in *P. aeruginosa*, *A. baumannii* and *S. aureus*. The inhibitory activity of the compounds was determined using EtBr accumulation assays in strains displaying varying level of efflux expression. The compounds resulted in a five to an eight-fold decrease in 90% effective concentration of tetracycline, chloramphenicol, and aztreonam towards *P. aeruginosa* strains. Furthermore, unlike other polyamines, which have been shown to disrupt bacterial membranes, no membrane destabilization was observed with the HTS hits from the study—this phenomenon can often lead to the selection of false positive EPIs. The compounds also demonstrated limited toxicity towards human cell lines, highlighting the potential of the scaffold for further development as anti-resistance agents.

A majority of these compounds have been the result of HTS ventures and bioassay-guided determination. Although these strategies have yielded several promising EPIs, they have often proven to be very laborious and expensive. Consequently, the use of bioinformatics and other in silico screening can provide a more efficient development route with minor costs. Mangiaterra et al. [68] performed in silico screening and molecular docking simulations against MexB protein to identify a series of MexAB substrates. The combination of the natural products, morelloflavone and pregnan-20-one-derivative synergistically reduced the MIC of ciprofloxacin four-fold, while significantly increasing the bactericidal properties of the antibiotic, in vitro. The compounds also showed the ability to increase intracellular EtBr (MexB substrate) concentration. The natural steroidal alkaloid, conessine, has been shown to significantly reduce the MICS (four to eight-fold) of cefotaxime, levofloxacin, tetracycline, erythromycin, novobiocin, and rifampicin in *P. aeruginosa* mutants (overexpressing MexAB-OprM). Moreover, synergistic actions, observed between other antibiotics and conessine in MexB deleted strains suggested inhibitory actions against other efflux systems.

In an in silico study, two non-antibiotic drugs, lanatoside C and daidzein, were validated to exhibit synergistic potential with antimicrobial MexB substrates, based on in vitro assays [69]. The correlation between in silico and in vitro tests in both studies indicates the promising implications of both the study method and the identified EPIs. Additional approaches include the employment of nanoparticles as EPIs to disrupt QS and rejuvenate the bactericidal effect of conventional antibiotics [70]. However, the potential implication of this approach has been impeded by the reactivity and toxicity of the nanoparticles.

### 5.2. Enterobacter Spp.

*Enterobacter* is a genus of facultative Gram-negative anaerobes that belongs to the *Enterobacteriaceae* family. These opportunistic pathogens commonly target immunocompromised hosts, who are implanted with IMD or on mechanical ventilation [71]. The genus has been implicated in a wide spectrum of nosocomial infections, namely urinary and respiratory tract infections. Subsequently, patients with a prolonged hospital stay, especially those in ICU are at increased risk of contracting associated infections. Given the ubiquitous nature of *Enterobacter*, infections can be acquired from both endogenous and exogenous source. However, most nosocomial infections are contracted endogenously from previously colonised patients who have received prior antibiotic therapy [71]. Among the different species of *Enterobacter*, *E. aerogenes* and *E. cloacae* are the two most frequently encountered pathogens in patients; although the former species has been speculated to belong to the *Klebsiella* genus [72]. *E. cloacae*, especially, currently accounts for 4%–5% of all nosocomial bacteraemia, pneumoniae and urinary tract infections [73]. Due to the species diversity of the genus itself, antimicrobial susceptibility varies widely within *Enterobacter*, however, both *E. cloacae* and *E. aerogenes* are almost uniformly resistant to ampicillin, cephalothin, cefoxitin and other *β*-lactam drugs, among others [71]. Both species have demonstrated the intrinsic ability to form biofilm, however, due to their relative prevalence among the *Enterobacter* genus, biofilm production has predominantly been studied in *E. cloacae* [74].

### 5.3. EPIs and Their Potential Role in Biofilm Disruption in Enterobacter Spp.

Concerning EPIs against *Enterobacter*, majority of the studies have been focused on *E. aerogenes* (*K. aerogenes*), due to the significance of RND pumps to their resistance profile. The AcrAB system in the pathogen shares a similar substrate specificity to that of other Gram-negative ESKAPEE pathogens [49]. For instance, similar to *A. baumannii*, both PAβN and NMP (Figure 2) exhibited a reversal of resistance of MDR *E. aerogenes* strains [49]. The aryl-piperazine derivatives restored susceptibility to various AcrAB-TolC substrates, including fluoroquinolones and norfloxacin [75]. Furthermore, resistant *E. aerogenes* isolates are also affected by the energy uncoupler, CCCP. Nevertheless, one of the main class of EPI investigated against *E. aerogenes* are quinoline derivatives.

**Figure 8:**
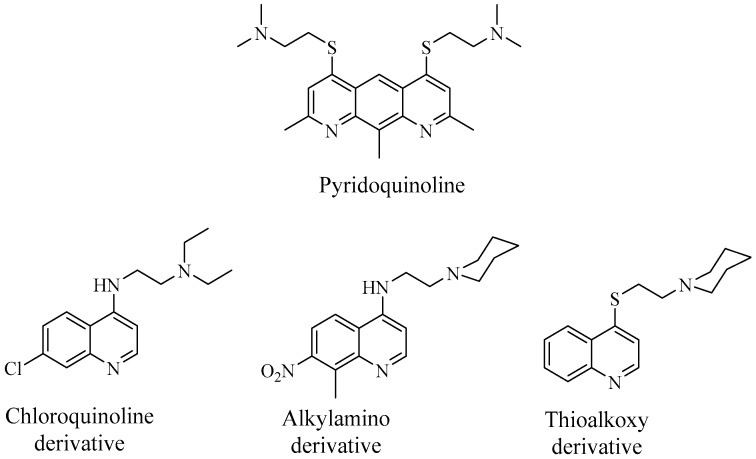
Quinoline derivatives investigated as EPIs against multi-drug resistant (MDR) *E. aerogenes* strains.

Quinoline derivatives has reportedly potentiated the activity of several structurally unrelated antibiotics in resistant *E. aerogenes* strains, overexpressing AcrAB-TolC pumps [76]. For instance, a chloroquinoline derivative with a diethylaminoethyl side chain (Figure 8) was found effective at modulating chloramphenicol activity in vitro with an 8-fold decrease in chloramphenicol MIC, as exhibited by efflux-overexpressing MDR strains [77]. Furthermore, the derivative increased intracellular accumulation of the antibiotic in the isolates, further determining the ability of the compounds to block AcrAB efflux activity. Additionally, pyridoquinoline derivatives, such as the one displayed in Figure 8, was found effective at restoring the activity of norfloxacin and fluoroquinolones on MDR *E. aerogenes* harbouring the AcrAB system, by outcompeting the antibiotics for the pump and resulting in an increased intracellular accumulation of the drugs [78]. Subsequent studies led to the development of 4-substitued alkoxy-, alkylamino and thioalkoxy-quinolines as potent EPIs. As per previous discussions, the alkoxy-quinoline derivative (905) exhibited the ability to potentiate the activity of tetracycline, norfloxacin and chloramphenicol in MDR strains of *K. pneumoniae* and *E. aerogenes* via AcrAB-TolC efflux inhibition [46]. Various alkyl-aminoquinolines have shown the capability to restore susceptibilities to several antibiotics in MDR *E. aerogenes* isolates exhibiting different resistance phenotype. In an in vitro study, alkyl-aminoquinoline derivatives conferred significant inhibitory activity at minimal concentration [79]. The analogue with piperidinoethyl side chain (Figure 8) was shown to increase the intracellular accumulation of chloramphenicol in MDR strains expressing efflux pump. In addition, the EPI also conferred a significantly higher level of inhibition than PA*β*N. Thioalkoxy-quinolines have also been found effective at increasing chloramphenicol sensitivity—in an in vitro study performed by Gallo et al. [80], 4-piperidinoethylthio-quinoline was shown to reduce MIC of the antibiotic by approximately 20-fold.

## 6. *Escherichia coli*

*E. coli* is a Gram-negative bacterium, part of the *Enterobacteriaceae* family that typically colonises the gastrointestinal tract of vertebrates. These commensal strains are rarely pathogenic and are part of the intestinal microflora, where they symbiotically benefit the host by outcompeting pathogenic bacteria. This is achieved through the production of bacteriocins and other mechanisms [37]. Nevertheless, some strains have exhibited pathogenic potential, causing extraintestinal infections (ExPEC) in humans including urinary tract infection (UPEC) and neonatal meningitis (NMEC) [37]. Notably, MDR *E. coli* strains exhibit higher resistance to antimicrobial agents in biofilm compared with planktonic cells [37].

### EPIs and Their Potential Role in Biofilm Disruption in E. coli

Small synthetic molecules with conjugated aromatic rings, such as phenyls, naphthalene, and indoles, which tend to be AcrAB-substrates, are of great interest in EPI research in *E. coli*. The addition of NMP have been shown to enhance susceptibility to several classes of antibiotics including levofloxacin, clarithromycin, EtBr and fluoroquinolones by increasing their intracellular concentration in *E. coli* strains overexpressing AcrB and AcrEF pumps [81]. The aryl-piperazine resulted in a ≥4-fold reduction in MIC of >50% of the isolates. However, NMP have faced limited success as EPIs due to its high toxicity at effective doses [82].

**Figure 9:**
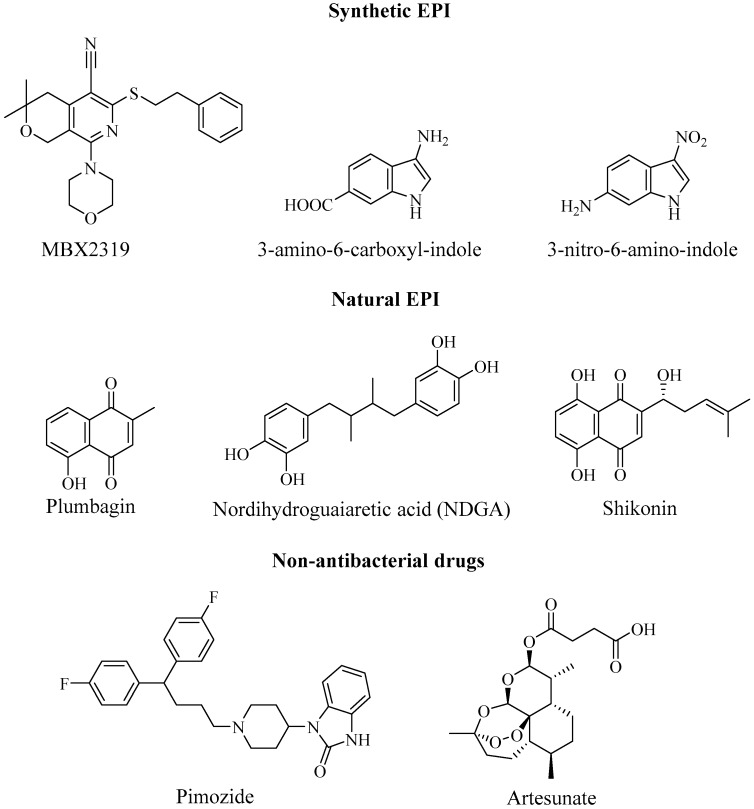
Potential EPIs directed against Resistance Nodulation Division (RND) pumps in *E. coli*.

The pyridopyrimidine analogue, D13-9001 (Figure 7), is the first inhibitor cocrystallised with the RND pumps, AcrB (*E. coli*) and MexB (*P. aeruginosa*) [83]. In addition to *P.* aeruginosa, the pyridopyrimidine derivative has also exhibited inhibitory activity against AcrB in *E. coli* strains—the EPI was found to potentiate erythromycin (AcrAB substrate) [83]. The proposed mechanism-of-action states that D13-9001 binds to the hydrophobic trap in the binding channel of the pumps with high affinity, sterically preventing the binding and transport of substrates. Despite an early promise from preclinical studies, the compound has yet to progress to the clinical stage [59]. Opperman et al. [84] identified a novel pyranopyridine, named MBX2319 (Figure 9), as a potent EPI of the RND efflux system in the *Enterobacteriaceae* family, namely *E. coli*. The EPI exhibited inactivity in mutants lacking the AcrAB-TolC pumps and inhibited the extrusion or accumulation of AcrAB substrates; thereby, ensuring its inhibitory activity against the pump. Moreover, MBX2319 reduced the MIC of ciprofloxacin, levofloxacin, and piperacillin versus 2-, 4- and 8-fold, respectively in WT *E. coli strains*, but was not active against AcrAB-TolC-deficient strains. In addition, the EPI also potentiated antibacterial AcrAB substrates in a range of other *Enterobacteriaceae* pathogens including *K. pneumoniae* and *Shigella flexneri* among others [84]. Similar to D13-9001, computational study indicated the presence of interaction of the EPI with the same “hydrophobic trap” of the AcrB pump, with the pyridine ring forming sterically-hindered ring-stacking interactions [85].

Zeng et al. [86] developed synthetic indole derivatives, based on the TolC structure in an attempt to inhibit the AcrAB-TolC pump. The indole structures, 3-amino-6-carboxyl-indole and 3-nitro-6-amino-indole (Figure 9) were reported to exhibit synergistic antimicrobial activity with chloramphenicol, tetracycline, erythromycin, and ciprofloxacin against *E. coli* strain overexpressing AcrAB pumps, decreasing their MIC 2-64 folds. The aforementioned AcrAB-TolC pump inhibitor, compound 1167 (Figure 5), has also demonstrated efflux inhibition in WT *E. coli* strains, with moderate potency [45].

There have been several reports of the ability of crude extracts from plants and other organisms to act as EPI and potentially reverse multi-drug resistance in *E. coli*, among other pathogens. Ohene-Agyei et al. [87] used in silico screening to identify plant-derived compounds, able to bind to and inhibit AcrB. Further in vitro analysis revealed the ability of plumbagin, nordihydroguaiaretic acid (NDGA) and shikonin (Figure 9) to increase susceptibility to several antibiotics through the inhibition of AcrB-mediated substrate efflux; hence displaying their potential to prevent biofilm formation.

Due to lack of progress in the antimicrobial drug pipeline, research has also focused on the implications of non-antibacterial drugs as EPIs. Phenothiazine derivatives, often used as psychotropic drugs, have previously been reported to inhibit the efflux systems of both Gram-negative and Gram-positive bacteria. Takács et al. [88] reported the in vitro analysis of four new phenothiazine derivatives for their inhibitory activity against efflux system in *E. coli*, *Salmonella enteritidis*, *E. faecalis* and *S. aureus*. The compounds exhibited significantly increased potency against Gram-positive bacteria, compared with Gram-negative. Phenothiazines, in addition to trifluoromethyl ketones, have also been shown to disrupt QS in *E. coli* via AcrAB-TolC inhibition [89] Another class of psychotropic drug, pimozide, was also reported to act as model EPI. Pimozide was shown to reduce the MIC of EtBr by 4-fold in EtBr accumulation assays, which was comparable to Pa*β*N [90]. The EPI was described as “narrow-spectrum” model due to its high substrate-specificity. Due to the toxicity of the plasma peak levels required for EPI activity, there is no possibility of using the drug clinically as an EPI. The anti-malarial drug, artesunate has been reported to enhance the antimicrobial effect of β-lactam drugs against *E. coli*, through the suppression of AcrAB-TolC efflux [91].

## 7. Future Prospective and Conclusion

The role of efflux pumps during biofilm formation has been extensively studied in the ESKAPEE pathogens, in the context of their upregulation and links to antibiotic resistance. The ESKAPEE pathogens are the leading cause of nosocomial infections, due to their intrinsic ability to develop resistance, and are increasingly becoming one of the greatest challenges of modern medicine. This ability has consequently rendered the majority of conventional antimicrobial treatments useless. EPIs could, therefore, play a major role in combating this global issue by reversing pathogenic resistance to the antibiotics. Studies have reported the ability of EPIs to reduce in vitro biofilm formation [92], which is one of the major contributing factors leading to phenotypic resistance/tolerance and multi-drug resistance. Their ability to directly affect biofilm formation and indirectly improve antibiotic activity makes them an attractive approach.

Ideally, EPIs would be able to inhibit a broad range of efflux pumps, from different families and found in a range of pathogenic species. However, this has proven to be a challenging task due to the wide diversity of the efflux pump systems in bacteria. Subsequently, the design of narrow-spectrum EPIs targeting specific efflux pump families could prove to be the best approach. Experimental methods including site-directed mutagenesis and X-ray crystallography are typically used to elucidate the functional assembly of efflux pump and EPI mechanism. Furthermore, X-ray crystallography coupled with in silico computational methods can prove to be a useful approach to understanding the underlying mechanism of this interaction, further providing a rationale for EPI design. However, these pumps are intrinsic multicomponent membrane proteins, and crystallisation of macromolecular complex structures have often proven to be extremely challenging [93]. This has hampered our ability to understand efflux-pump function and define their substrate profile, consequently, preventing us from designing effective, target-specific EPIs. A possible strategy for the development of EPIs could be the screening of pre-approved drugs; thereby, circumventing the risks associated with developing new chemical entities. However, many drugs will be toxic at the concentrations required to inhibit efflux, as seen with reserpine, verapamil and PA*β*N among others [37].

Despite current efforts, our knowledge of the exact roles of the efflux systems in biofilm formation is very limited. Most studies use deletions of efflux pumps and/or their regulators and infer the role of the pump in biofilm formation from studies with these strains. However, deleting efflux pump components will have pleiotropic effects, where other pumps or membrane proteins compensate for the loss of components in the bacterial membrane by altering their expression levels. This necessitates a different approach to study the role of the pumps and effect of EPI during biofilm formation. There is no direct evidence for how inhibition of a specific efflux pump affects biofilm formation. For example, we do not know what the non-antibiotic, efflux substrates are that are being exported by the majority of pumps and cannot define how these influence biofilm formation. The development of highly effective EPIs coupled with mass spectrometry and metabolomics studies to identify cognate efflux pump substrates influenced by inhibition, might be an effective means to understand the phenotypes generated.

The clinical translation of these promising EPIs remains problematic due to off-target effects, relatively low potency, poor PK/PD and human cell toxicity. No study has investigated the role of the pumps using in vivo biofilms, which has made it difficult to assess their absolute role in biofilm infections. It would not be surprising if there are differences in the role of efflux pumps in mediating biofilm formation/disruption under physiologically relevant settings. If these issues can be overcome, then it may be possible to advance EPIs to the clinic with properties that inhibit biofilm formation and, simultaneously, potentiate the action of antibiotics. Such therapeutics would be highly valuable in developing new treatments for the management of biofilm forming MDR pathogens, especially those from the ESKAPEE group.

## Figures and Tables

**Table 1:** Clinical manifestations of biofilm-related infections (mostly ESKAPEE pathogens (Enterococcus faecium., Staphylococcus aureus, Klebsiella pneumoniae, Acinetobacter baumannii, Pseudomonas aeruginosa, Enterobacter spp. and Escherichia coli) infections).

Biofilm Infections Site	Clinical Manifestations	Bacteria Species	References
Endocarditis (heart)	Patients with or without prosthetic heart valves or pacemaker, who have intermittent fever and bacteraemia with an identical pathogen.	*Streptococcus* species *Enterococcus* species *Staphylococcus aureus* Coagulase-negative *Staphylococci*	[19] [20] [21] [22]
Biofilm infection in CF/chronic obstructive pulmonary disease (COPD) (lungs)	Presence of bacteria detected in sputum.	*Pseudomonas aeruginosa*	[23] (CF) [24] (COPD)
Intravenous catheter	Patients with central venous catheter or haemodialysis catheter, who exhibit recurrent bacteraemia with an identical pathogen.	*Escherichia coli* Coagulase-negative *Staphylococci* *Klebsiella pneumoniae* *Pseudomonas aeruginosa* *Enterococcus aerogenes*	[25] [26] [25] [25] [25]
Orthopaedic infections (musculoskeletal system)	Local chronic pain and sign of prostheses loosening in patients with joint prostheses or orthopaedic fixation devices.	*Staphylococcus aureus* Coagulase-negative *Staphylococci*	[27] [28]
Urinary catheter	Recurrent urinary tract infections with the same pathogens in patients’ urinary catheter.	*Klebsiella pneumonia* *Acinetobacter baumannii* *Candida* species *Enterococcus* species *Enterobacter* species	[29] [30] [31] [32] [33]
Chronic wounds	Patients with chronic wounds suffering from recurrent wound infections.	*Staphylococcus aureus* *Pseudomonas. aeruginosa*	[34] [34]
